# Training the Trainers in Ultrasound-guided Access to Improve Peripheral Intravenous Catheter Placement among Children Presenting for Anesthesia

**DOI:** 10.1097/pq9.0000000000000406

**Published:** 2021-05-05

**Authors:** Vikas N. O’Reilly-Shah, Amber Franz, Cornelius B. Groenewald, Michael Collins, Lance S. Patak

**Affiliations:** From the Department of Anesthesiology and Pain Medicine, University of Washington School of Medicine, Seattle Children’s Hospital, Seattle, Wash.

## Abstract

Factors predicting difficult peripheral intravenous (PIV) catheter placement in children are known. Ultrasound guidance can decrease the number of attempts required for difficult PIV placement. However, the uptake of this technique among attending anesthesiologists at our institution remained low. This study aimed to reduce the incidence of PIV placement requiring greater than 3 attempts and reduce time to PIV placement by providing training in ultrasound guidance.

**Methods:**

We implemented an evidence-based difficult IV algorithm and ultrasound-guided PIV placement training for attending pediatric anesthesiologists at a tertiary academic pediatric institution. The algorithm outlined risk factors for difficult IV access, established a goal of 3 or fewer attempts, and recommended early ultrasound use after 1 unanticipated attempt and the first attempt for anticipated difficult IV. Group sessions, including instruction and simulated practice, preceded a period of individual training in the operating room using a punch card to monitor each trainee’s progress while also serving to motivate continued engagement.

**Results:**

We performed a cross-sectional analysis of consecutive cases from December 1, 2015, to September 30, 2019, comparing a 22-month baseline period (n = 12,581) with the training period (n = 6,725) and the following year (n = 6,557). Cases requiring more than 3 attempts decreased from 4.0% to 2.7% overall and from 10% to 6.2% among patients 24 months or less of age. The time required to establish PIV access was unchanged. Factors associated with increased attempts were identified.

**Conclusions:**

Implementing a difficult IV algorithm and training among attending pediatric anesthesiologists in ultrasound-guided PIV placement reduced attempts but not the time required to establish PIV access.

## Abstract 2

Supplemental Digital Content is available in the text.

## INTRODUCTION

Approximately, 6 million children undergo surgery in the United States each year.^1^ For most elective pediatric anesthetics, an inhalational agent (eg, sevoflurane) is used to induce general anesthesia followed by peripheral intravenous (PIV) access to administer medications or fluids. The time during which the child is anesthetized without PIV access is fraught with difficulties or emergencies such as airway obstruction, laryngospasm, or arrhythmias. These issues are more challenging to address without PIV access.

Predictors of difficult intravenous access (DIVA) are known and include younger age, higher body mass index (BMI), medical comorbidities, and darker skin pigmentation.^2–6^ Studies from the emergency room and perioperative settings reveal that DIVA occurs in approximately 5% of children, leading to poorer patient experience, delays in care, and increased cost.^3,6–9^ Clinical care pathways directing earlier use of ultrasound can reduce the number of PIV attempts, yielding an improved patient experience.^10–14^ Also, using ultrasound in DIVA can reduce the need for central venous catheter placement, mitigating risks attendant to the placement and use of those catheters (eg, pneumothorax and line infection).^15^ In anesthetized toddlers, ultrasound use improves first-pass success, potentially at the cost of increased time to placement in the aggregate.^16^

Accompanying the advent of ultrasound use over the past decade is training gaps with increasing time since completing postgraduate training.^17,18^ A pragmatic limitation on using ultrasound for DIVA is a lack of provider training and comfort. The present work reports the results of a quality improvement project designed to increase anesthesiologists’ familiarity with using ultrasound for PIV access and drive earlier use of ultrasound guidance for PIV placement for both anticipated and unexpected DIVA.

This project was motivated by an anecdotal but troubling pattern of complaints from parents and surgeons regarding the number of PIV attempts and the length of time to PIV placement in notable outlier cases, particularly among repeat patients with known DIVA. Baseline departmental survey data (**Tables S1–S2, Supplemental Digital Content 1,** http://links.lww.com/PQ9/A253) among all 55 attending anesthesiologists on staff showing: (1) a majority (67%) did not use ultrasound even once per week for PIV placement; (2) most (70%) would either not consider using ultrasound or would wait until the third attempt or later; (3) a trend toward those with more than 10 years of experience having a lower self-rated skill and higher self-reported rate of never turning to ultrasound for PIV/DIVA; and (4) the top tier confidence level using ultrasound for DIVA averaging only 56.6/100 or equivalently 5.7 on a 10-point scale. A priori-defined quality improvement interventions included: (1) anesthesiologist education/simulation; (2) development of a DIVA algorithm to help reduce PIV attempts; and (3) a “punch card” to monitor progress and motivate hands-on training with ultrasound. We hypothesized that this program of interventions would achieve the following primary and secondary aims, respectively (1) reduce the number of cases in which providers made more than 3 PIV attempts and (2) improve the efficiency of PIV placement, defined as a reduction of time between “in-room” and PIV placement.

## METHODS

### Ethical Considerations

Our hospital’s Institutional Review Board (IRB STUDY00000407) reviewed and approved this study, including a waiver of written informed consent. The IRB raised no specific ethical concerns nor reported any conflicts of interest. This article adheres to SQUIRE 2.0 reporting guidelines.^19^

### Context

Our institution is an academic, tertiary care, pediatric hospital. At the start of the intervention period (see below), 55 attending anesthesiologists were on staff, including 36 full-time staff (at least 80% clinical effort) and four part-time staff (less than 50% clinical effort). Though the focus of the present work is on attending anesthesiologists at our institution, anesthesia is provided at our hospital either with a solo attending anesthesiologist or under a care team model, including attending anesthesiologists, trainees, and certified registered nurse anesthetists. Our process for a typical inhalational induction involves a safety sign-out in our preoperative holding area, parental presence on induction rather than oral anxiolysis, preinduction in-room identification, and placement of monitors before induction (pulse oximeter at minimum, dependent on patient cooperation). There is variable presence and assistance from trainees, anesthesia technologists, and operating room staff in the performance of these tasks. The approach to the documentation of PIV placement involves a single click to move the event from a macrogenerated list of presented events. Notably, there was a significant disruption to the workflow that occurred due to operating room closures for updates and rehabilitation related to infection control issues during the postintervention period.^20^ This provided an opportunity to highlight the impact of this workflow disruption on DIVA.

### Interventions

Our interventions targeted modifying our culture and approach to PIV placement amongst attending anesthesiologists, not towards specific cases of anticipated (or unanticipated) DIVA. There were 2 significant elements to this intervention. First, we developed and disseminated a DIVA algorithm identifying risk factors for DIVA and outlining a goal of no greater than 3 attempts to establish PIV access with or without ultrasound (**Figure S1a, Supplemental Digital Content 1,** http://links.lww.com/PQ9/A253).^6^ This algorithm did not explicitly state DIVA predictors, as these are well established (younger age, higher BMI, increased comorbidities). Instead, the algorithm focused on providing a target (IV access within 3 attempts) and encouraged earlier ultrasound use. We based this algorithm on a previous study published by our group, finding that DIVA occurred in 5.3% of anesthetics in our practice and was associated with several risk factors and prolonged time to surgical incision, notably following the third attempt.^6^ Second, we implemented a training program for attending anesthesiologists, consisting of 2 group learning sessions with simulation practice using a training model (Blue Phantom, CAE Healthcare Sarasota, Fla.) and 1:1 training and mentorship in the operating room using the technique described by Gopalasingam et al,^16^ as this was the most common technique employed by those proficient in ultrasound-guided PIV placement at our institution. A volunteer group of anesthesiologists known to be proficient in ultrasound-guided PIV access (defined as greater than 90% chance of successful IV placement using ultrasound) before the intervention was sought to act as trainers. These anesthesiologists were the people others called when they needed help with DIVA. Anesthesiologists interested in receiving the training (“trainees”) were issued a punch card after the group learning sessions (**Figure S1b, Supplemental Digital Content 1,** http://links.lww.com/PQ9/A253) to track progress in the operating room. Once an attending achieved first-pass ultrasound-guided PIV success on 10 patients (ten punches), they passed from the trainee pool to the trainer pool. We chose this threshold based on expert consensus amongst the initial group of trainers; to our knowledge, there were no published data on learning curves in this area. Participation in the program was voluntary, as was adherence to the suggested algorithm. No formal assessment of punch card effectiveness was performed.

### Study of the Interventions

This study is a secondary cross-sectional analysis of 25,863 consecutive pediatric anesthesia cases (age 18 years and younger) from December 1, 2015, to September 30, 2019. The data were partitioned into three periods, representing the Baseline period (December 1, 2015, to October 31, 2017, before any intervention), Training period (November 1, 2017, to September 30, 2018, during which the simulation session and peer-teaching occurred), and postintervention period (October 1, 2018, to September 30, 2019, following effective peer-teaching completion). Routinely documented clinical data were extracted as a limited dataset from the electronic medical record (Cerner Surginet Anesthesia Database, Kansas City, Mo.) and an enterprise data warehouse maintained by the hospital system. The study population included all cases where a PIV placement started in the operating room. We excluded: patients older than 18 years of age, cases with incomplete data, cases with the American Society of Anesthesiologists-Physical Status (ASA-PS) 5, and cases where a PIV was present at induction. Some patients may have had repeated surgeries during the study period.

### Measures

Anesthesiology providers routinely documented PIV placement time, location, size, a subjective binary indication of difficulty, and the number of attempts required for successful placement. Starting in February 2018, providers began documenting ultrasound use for placement. This study’s primary outcome was the number of cases in which PIV placement required more than three attempts. The secondary outcome was the period between entering the operating room (“in-room”) and PIV placement. Patients were separated by age since younger children often pose more difficulty for PIV placement.^21^

We tracked the number of anesthesiologists who self-selected to complete the punch card (10 first attempt successes with ultrasound-guided PIV). This metric served as an indicator of training compliance and as a process measure. For a balancing measure, we looked to see if ultrasound use increased PIV placement time, which would be detrimental to patient safety and efficiency. Time could increase if providers were waiting for an ultrasound to be delivered to their operating room or if providers were not skilled enough to efficiently execute ultrasound-guided PIV placement.

### Covariates

Covariates included patient age, sex, ASA-PS status, BMI, and emergency case status. BMI was binned and treated as a categorical variable due to the nonlinearity associated with the logit for the regression analysis.

### Statistical Analysis

Analyses were performed using R version 3.5.2 (R Core Team, Vienna, Austria) within RStudio platform v1.1.423 (R Studio Team, Boston, Mass.). Baseline demographic variables were compared using chi-squared tests for categorical variables (age, ASA-PS, emergency case, and reported difficult) and Wilcoxon rank-sum tests for the non-normally distributed BMI. Control charts were generated using the R package “qicharts2.”

We developed U-type statistical process control charts to assess the secular trend and change due to the intervention in the rate of cases in which more than three attempts at PIV access occurred. As a sensitivity analysis, we developed a multivariable logistic regression model to determine associations between cases requiring greater than three PIV attempts and baseline risk factors with univariate association *P* < 0.2 (age, BMI, and ASA-PS) and exposure to the intervention period. The Hosmer–Lemeshow test and c-statistics were calculated. We generated Kaplan–Meier plots of the period between “in-room” time and PIV placement time, segregated by the intervention period. The “in-room” to PIV placement time was winsorized using the “psych” package R (trim = 0.005) for the final Kaplan–Meier plot to mitigate the impact of outliers on the Figure; sensitivity analyses with and without winsorization showed no difference in the result. Quantile regression for testing differences at the 95th percentile was performed using the “quantreg” package.

## RESULTS

**Fig. 1.**
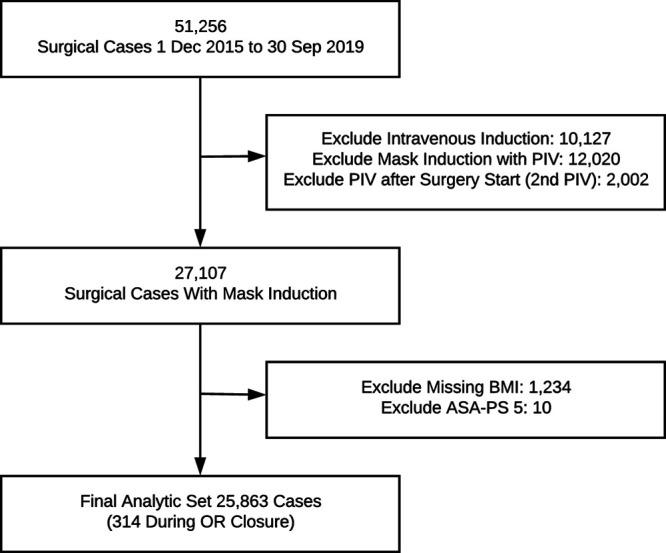
Case flow diagram for included cases.

Four attending anesthesiologists agreed to act as trainers at the start of the intervention period. Thirty-six anesthesiologists (65%) attended group learning sessions, 23 (42%) received punch cards, and 16 (29%) self-selected to complete full training by performing 10 successful ultrasound-guided PIV placements, indicating low compliance with the full training program. The case inclusion flow diagram is shown in Figure 1, and baseline demographic information for included cases (Table 1) is stratified by the intervention period. There were statistically significant differences in patient age and ASA-PS between the 3 periods, with a small trend toward older patients and a substantive trend towards increased ASA 2 and 3 patients in the training and postintervention periods.

**Table 1.** Case Demographic Information Stratified by the Study Period

		Period			
	Level	Baseline	Training	Postintervention	*P*
n		12,581	6,725	6,557	
Age category (%)	Younger than 30 d	47 (0.4)	20 (0.3)	28 (0.4)	<0.001
	1–3 mo	346 (2.8)	149 (2.2)	170 (2.6)	
	4–6 mo	478 (3.8)	240 (3.6)	237 (3.6)	
	7–9 mo	460 (3.7)	225 (3.3)	208 (3.2)	
	10–11 mo	309 (2.5)	134 (2.0)	139 (2.1)	
	12–23 mo	1,340 (10.7)	692 (10.3)	655 (10.0)	
	2–4 y	2,617 (20.8)	1,483 (22.1)	1,369 (20.9)	
	5–7 y	2,124 (16.9)	1,082 (16.1)	1,088 (16.6)	
	8–10 y	1,955 (15.5)	1,019 (15.2)	936 (14.3)	
	11–15 y	2,184 (17.4)	1,214 (18.1)	1,286 (19.6)	
	16+ y	721 (5.7)	467 (6.9)	441 (6.7)	
BMI (median [IQR])		16.80 [15.43, 18.99]	16.86 [15.38, 19.20]	16.91 [15.40, 19.27]	0.086
ASA-PS (%)	1	3,247 (25.8)	1,695 (25.2)	1,365 (20.8)	<0.001
	2	5,486 (43.6)	2,880 (42.8)	2,892 (44.1)	
	3	3,526 (28.0)	1,973 (29.3)	2,113 (32.2)	
	4	322 (2.6)	177 (2.6)	187 (2.9)	
Emergency case (%)	No	12,517 (99.5)	6,703 (99.7)	6,534 (99.6)	0.106
	Yes	64 (0.5)	22 (0.3)	23 (0.4)	
Ultrasound used (%)	No	No data	Partial data	6,019 (91.8)	—
	Yes	No data	Partial data	538 (8.2)	
Reported as difficult PIV (%)	Not Difficult	11,858 (94.4)	6,324 (94.1)	6,191 (94.4)	0.589
	Difficult	704 (5.6)	398 (5.9)	364 (5.6)	
More than 3 attempts (%)	3 or less	12,082 (96.0)	6,544 (97.3)	6,380 (97.3)	<0.001
	More than 3	499 (4.0)	181 (2.7)	177 (2.7)	

**Fig. 2.**
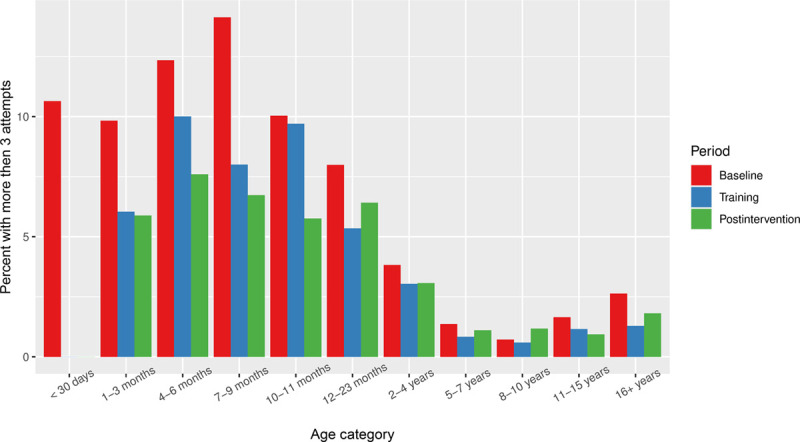
Percentage of cases where more than 3 attempts were made for peripheral intravenous catheter access, stratified by the study period.

**Fig. 3.**
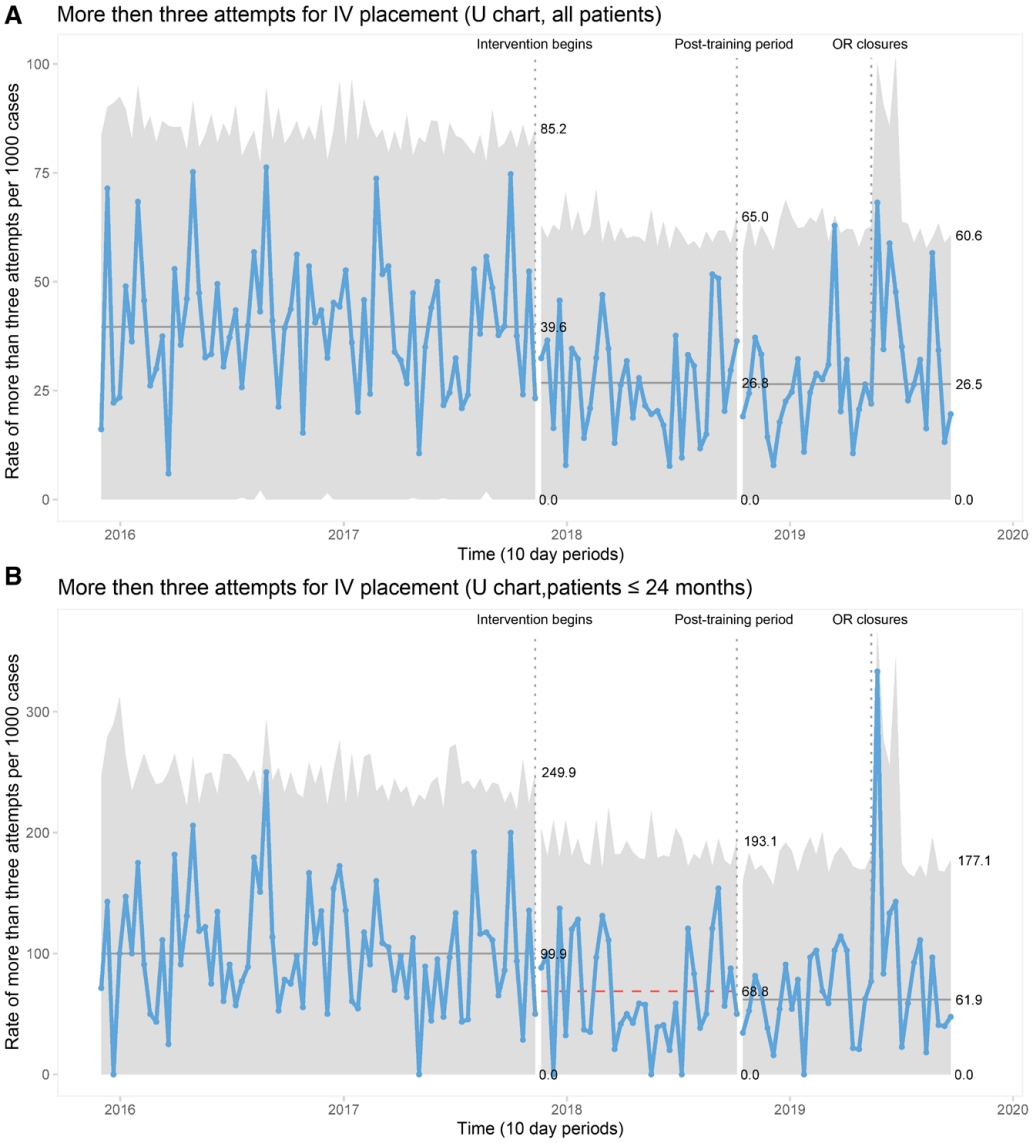
Control charts demonstrating the rate of requiring more than three peripheral intravenous catheter attempts per 1,000 cases for (A) all patients and (B) patients less than 24 months of age. The timing of operating room closures is noted.

Figure 2 illustrates the unadjusted rate of cases in which more than 3 PIV attempts were required, stratified by intervention period and age category. Notably, the highest rates of cases requiring greater than three attempts were primarily among patients less than two years of age—patients less than 4 years of age were above the baseline rate. Figure 2 demonstrates reductions in the rate of greater than 3 attempts for nearly every age category examined on an unadjusted basis. Figure 3 demonstrates the control chart for all patients and patients less than 2 years of age. This chart demonstrates that there was no secular trend toward reductions in the rate of greater than 3 attempts before the intervention.

Furthermore, this control chart demonstrates a stable process with sustained reductions in the rate of greater than 3 attempts through the training and postintervention periods. Cases requiring more than three PIV attempts decreased from 4.0% to 2.7% overall and from 10% to 6.2% among patients aged 24 months or less. Finally, the control chart demonstrates a substantial impact on PIV placement that accompanied disruptions to workflow associated with OR closures as a source of special cause variation, as evidenced by single points outside the upper control limit. This finding may be because only the most urgent cases and sickest children were scheduled at this time.

**Fig. 4.**
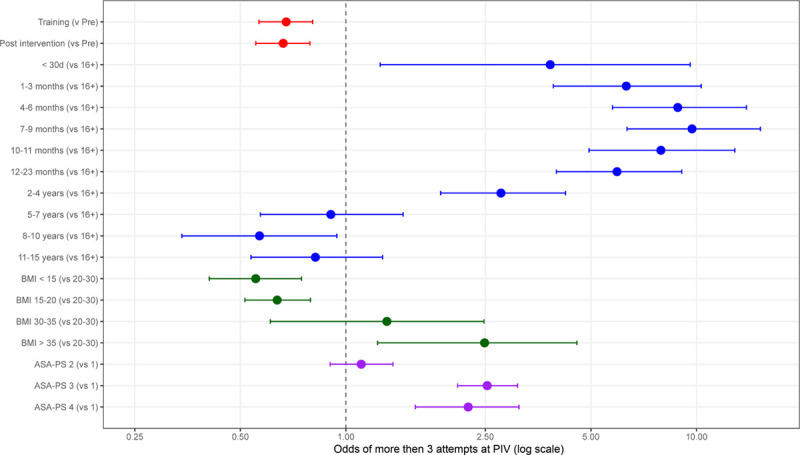
Multivariable logistic regression model demonstrating the adjusted association of the training and postintervention periods with reduced odds of a case requiring more than 3 attempts at peripheral IV. Increasing BMI and increasing ASA-PS were associated with a greater risk of requiring greater than 3 attempts.

The results of a multivariable logistic regression model are provided in Figure 4 (detailed values in **Table S3, Supplemental Digital Content 1,** http://links.lww.com/PQ9/A253), demonstrating that on an adjusted basis, there was an association of the training and postintervention periods with reduced odds of a case requiring more than three attempts at PIV access (training period odds ratio: 0.68 [0.57–0.8]; postintervention odds ratio: 0.66 [0.55–0.79]). Increasing BMI and increasing ASA-PS also posed a greater risk of requiring greater than 3 attempts.

**Fig. 5.**
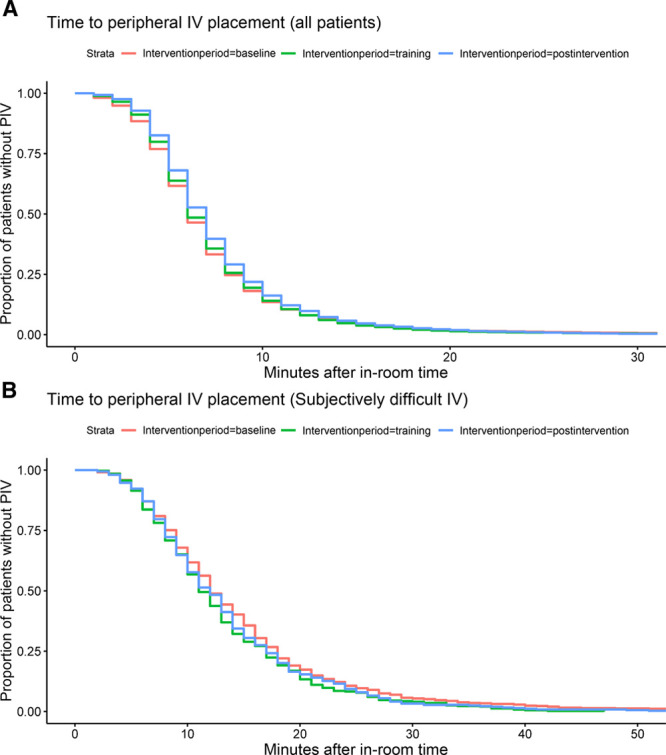
Kaplan–Meier plot demonstrating time between room entry and peripheral intravenous catheter placement stratified by (A) intervention period and (B) for the subset of patients characterized as subject difficult to place the peripheral intravenous catheter.

Figure 5 demonstrates the Kaplan–Meier analysis of time from “in-room” to PIV placement for all patients and patients subjectively characterized as DIVA. This analysis demonstrated that there was a statistical but not practical difference between the periods (Fig. 5A), with the 50th percentile of 6–7 minutes for each of the periods (*P* < 0.001). There were also statistical but not practical differences amongst patients subjectively characterized as difficult (Fig. 5B), with the 50th percentile of 11–12 minutes for each of the periods (*P* < 0.001). Quantile regression demonstrated that at the 95th percentile, there was a statistical (*P* < 0.001) but not practical difference of 1 minute between baseline and postintervention for the complete cohort, and no statistical differences between periods in the cohort of patients characterized as difficult. As above, there was no increase in time from in-room to PIV placement after implementing our intervention, our stated process measure.

## DISCUSSION

### Summary

We found that a quality improvement intervention aimed at anesthesiologist education, development of a DIVA algorithm, and using a punch card training-the-trainers approach with ultrasound-guided PIV access demonstrated an overall significant decrease in the rate of cases with 3 or more attempts at PIV placement. In contrast, we did not find that the intervention decreased time to PIV placement.

### Interpretation

Ultrasound guidance may help reduce vascular access attempts and, in some cases, reduce the time to successful PIV catheter placement.^11–14,16,22^ Ultrasound guidance for vascular access in emergency departments is increasingly common.^7,9,12,13,22–24^ A randomized controlled trial of 167 children presenting to the emergency room with DIVA, comparing ultrasound-guided PIV to palpation methods, found improved first-pass success, fewer attempts, and faster readiness.^25^ These findings may not hold in children without DIVA presenting to the emergency department.^26^

Our findings extend this work by demonstrating that an algorithm and training can substantially impact the number of PIV attempts. Excess time spent with DIVA contributes to patient safety risk, operating room inefficiency, variability in care, and reduced patient/parent satisfaction. Though we observed no difference in time to achieve IV access, our analysis lacked data on ultrasound use before February 2018. Efficiency gains seen with ultrasound use in the emergency room will be impacted by the time required to deliver and set up ultrasound machines on a just-in-time basis. We recommend establishing a process, whereby ultrasound machines are physically present at induction for all cases where a difficult IV is anticipated (due to history of DIVA or based on patient characteristics). Patients and families benefit from reduced PIV attempts, facilities, and providers benefit from improved patient satisfaction and perceived competence and safety.^23^

We emphasize that our implementation was pragmatic. Participation in training was voluntary, as was adherence to the DIVA algorithm. This approach avoided conflict or resentment among participating staff at the potential cost of reduced efficacy. Simply highlighting this as an area for improvement in the department encouraged anesthesiologists to be more thoughtful about their approach. Although not formally assessed, the punch card anecdotally served to motivate the trainee pool by promoting the sharing of techniques and success and encouraging friendly peer competition. Indeed, our improvements in mitigating excess PIV attempts were observed despite the 29% rate of participant punch card completion. Only attending anesthesiologists were specifically trained; however, trainees, certified registered nurse anesthetists, and nurses also often make the initial attempts at establishing PIV access in our context. For this reason, we specifically did not attempt to analyze those that completed training versus those that did not. Our approach demonstrated success with practical implementation that would be expected to translate to efforts at other institutions.

The temporary operating room closures (Fig. 3) disrupted workflow in several ways: case volumes were diminished; a major proportion of cases were performed in ad hoc off-site locations; physical access to normal operating room locations and the anesthesia workroom, was limited. We attribute the observed adverse impact on DIVA management to these factors.

### Limitations

As mentioned, we were unable to assess changes in ultrasound utilization as baseline data were not available. Differences were observed in patient demographics between the baseline and intervention periods. As with any retrospective analysis, differences in unobserved confounders could also present a source of bias; the lack of routinely recorded data on darker skin pigmentation deserves mention in this context. We do not use a formal DIVA scoring at our center in the perioperative setting, preventing comparison between cohorts of objectively identified DIVA patients. A scoring system to assess the risk of DIVA has been validated in the emergency department setting.^2^ However, this system does not include higher ASA-PS scores and higher BMI that are known predictors.^6^ We did not monitor the availability of ultrasound machines or time to retrieve if one was not readily available or the impact ultrasound readiness may have had on placement time using ultrasound guidance. Some patients may have had more than one procedure during the study period; prior knowledge of DIVA may have influenced the approach to subsequent attempts.

It may have been counterproductive to monitor adherence to the DIVA algorithm in our setting; independence and autonomy are well-established values in our department. Further improvements may or may not be seen if ultrasound training were required, if competence metrics were instituted, and/or if compliance with the difficult IV algorithm was tracked and enforced. Audit and feedback dashboards of IV attempts for patients with known risk factors for DIVA may also drive further improvements; nudge interventions signaling the presence of risk factors to providers may also prove effective. These are all areas worthy of future investigation.

## CONCLUDING SUMMARY

This 4-year longitudinal secondary cross-sectional analysis of 25,863 consecutive pediatric anesthesia cases demonstrates consistency across several predicted variables for DIVA, including increasing ASA-PS, increasing BMI, and age younger than 24 months. Training with ultrasound-guided PIV placement and implementing a goal-directed, evidence-based DIVA algorithm appeared to reduce the number of attempts at PIV placement with DIVA at our institution. Availability of ultrasound machines, number of providers competent in the skill, and degree of compliance to an established DIVA algorithm may have played a role in reducing the number of attempts to PIV placement for DIVA at our site. Anesthesiology departments should focus not just on ultrasound availability but also on competency and awareness of the risk factors that contribute to DIVA if they choose to implement a similar intervention.

## DISCLOSURE

The authors have no financial interest to declare in relation to the content of this article.

## ACKNOWLEDGMENTS

The authors are grateful to the informatics team at Seattle Children’s Hospital, Gayle Garson and Amber Yun, for extracting a series of data collections from our electronic medical record. The authors also thank and acknowledge Dr. John Bastien for updating our peripheral intravenous catheter documentation within our electronic medical record so that the appropriate data related to difficult IV management could be captured and later extracted.

## Supplementary Material


