# Supplementary material for: Training the Trainers in Ultrasound-guided Access to Improve Peripheral Intravenous Catheter Placement among Children Presenting for Anesthesia
Source: Pediatr Qual Saf. 2021 May 5;6(3):e406. doi: 10.1097/pq9.0000000000000406 (PMC8104256; doi:10.1097/pq9.0000000000000406)
Supplement: Supplementary file 1 [file pqs-6-e406-s001.pdf]

SDC Figure 1: (a) Difficult IV algorithm posted in all anesthetizing locations, (b) Punch cards issued to providers.

(a)

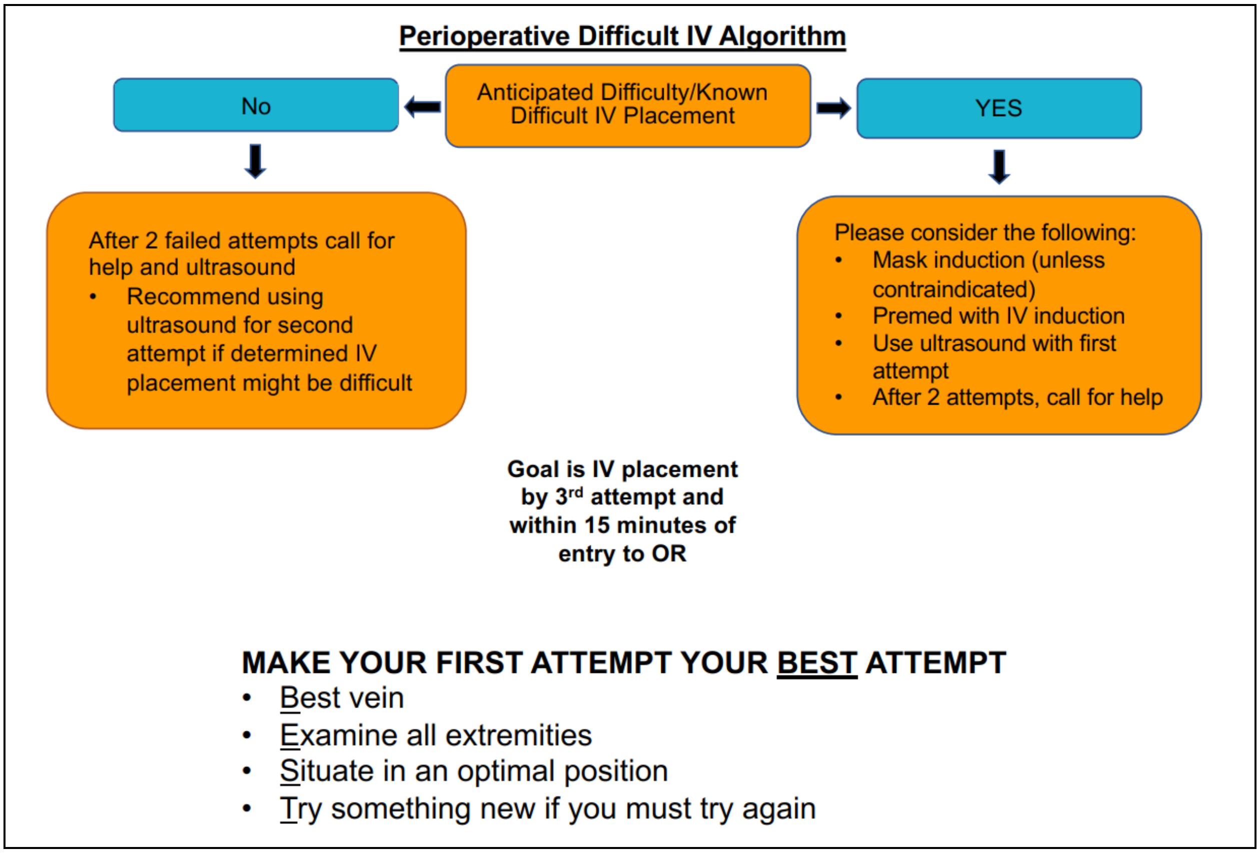

(b)

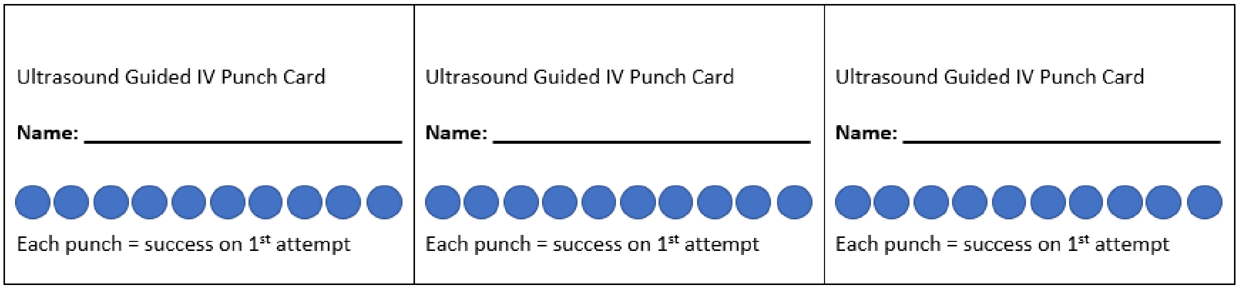

SDC Table 1: Results of a pre-survey prior to an ultrasound-guided IV workshop grouped by years of experience showing number of attending anesthesiologists reporting (a) ultrasound use at least once per week, (b) frequency of ultrasound use for peripheral IV, (c) average aggregate self-described expertise (scale: 0-100; higher rating for greater expertise) with ultrasound-guided IV placement.

Years experience	*I don't use an ultrasound for PIV.*		*Use an ultrasound at least once per week*		Self-Reported Level of Expertise median (interquartile range)
	Disagree	*Agree*	*No*	*Yes*	
< 3	9 (100%)	0 (0%)	4 (44.4%)	5 (55.6%)	66 (50-72)
3 to 5	3 (75%)	1 (25%)	3 (75%)	1 (25%)	43 (39-51)
5 to 10	14 (82.4%)	3 (17.6%)	11 (64.7%)	6 (36.3%)	50 (22-75)
10 to 15	8 (61.5%)	5 (38.5%)	10 (76.9%)	3 (23.1%)	30 (1-50)
> 15	8 (66.7%)	4 (33.3%)	9 (75%)	3 (25%)	23.5 (0-53)

SDC Table 2: Results of a pre-survey prior to an ultrasound guided IV workshop showing number of peripheral IV attempts before calling for ultrasound.

Number of Attempts Before Calling for Ultrasound	Number of Respondents
0	3 (5.5%)
1	2 (3.6%)
2	11 (20.0%)
3	19 (34.5%)
4	5 (9.1%)
5	2 (3.6%)
6	1 (1.8%)
I don't use ultrasound	12 (21.8%)

SDC Table 3: Multivariable logistic regression model demonstrating the adjusted association of the training and post-intervention periods with reduced odds of a case requiring more than 3 attempts at peripheral IV.

	Odds Ratio Estimate (95% CI)	*p-value*
Training (v Pre)	0.68 (0.57 - 0.8)	1.26E-05
Post Intervention (vs Pre)	0.66 (0.55 - 0.79)	5.54E-06
< 30 days (vs 16+)	3.83 (1.25 - 9.6)	0.008417939737
1-3 months (vs 16+)	6.31 (3.91 - 10.32)	9.22E-14
4-6 months (vs 16+)	8.84 (5.76 - 13.88)	2.01E-22
7-9 months (vs 16+)	9.71 (6.34 - 15.22)	2.02E-24
10-11 months (vs 16+)	7.92 (4.94 - 12.86)	1.92E-17
12-23 months (vs 16+)	5.93 (3.98 - 9.08)	2.07E-17
2-4 years (vs 16+)	2.77 (1.86 - 4.23)	1.06E-06
5-7 years (vs 16+)	0.91 (0.57 - 1.46)	0.6778299128
8-10 years (vs 16+)	0.57 (0.34 - 0.94)	0.02821761346
11-15 years (vs 16+)	0.82 (0.54 - 1.27)	0.3621689383
BMI < 15 (vs 20-30)	0.55 (0.41 - 0.75)	0.0001272616517
BMI 15-20 (vs 20-30)	0.64 (0.52 - 0.79)	3.84E-05
BMI 30-35 (vs 20-30)	1.31 (0.61 - 2.48)	0.4470657884
BMI > 15 (vs 20-30)	2.49 (1.23 - 4.56)	0.005890744969
ASA-PS 2 (vs 1)	1.11 (0.9 - 1.36)	0.3381611225
ASA-PS 3 (vs 1)	2.53 (2.08 - 3.09)	2.33E-20
ASA-PS 4 (vs 1)	2.23 (1.58 - 3.12)	3.59E-06

Increasing BMI and increasing ASA-PS were associated with a greater risk of requiring greater than 3 attempts. Hosmer-Lemeshow test (g = 19, p = 0.259) showed no evidence of poor fit; c-statistic (ROC-AUC) = 0.773.
